# Insectivorous bats integrate social information about species identity, conspecific activity and prey abundance to estimate cost–benefit ratio of interactions

**DOI:** 10.1111/1365-2656.12989

**Published:** 2019-04-22

**Authors:** Daniel Lewanzik, Arun K. Sundaramurthy, Holger R. Goerlitz

**Affiliations:** ^1^ Acoustic and Functional Ecology Max Planck Institute for Ornithology Seewiesen Germany; ^2^ Faculty of Biology Ludwig‐Maximilians‐University München Germany

**Keywords:** bats, biosonar, competition, eavesdropping, echolocation, heterospecific interactions, heterospecific recognition, information transfer

## Abstract

1. Animals can use inadvertent social information to improve fitness‐relevant decisions, for instance about where to forage or with whom to interact. Since bats emit high‐amplitude species‐specific echolocation calls when flying, they provide a constant flow of inadvertent social information to others who can decode that acoustic information. Of particular interest is the rate of feeding buzzes—characteristic call sequences preceding any prey capture—which correlates with insect abundance.
2. Previous studies investigating eavesdropping in bats yielded very different and in part contradictory results likely because they commonly focused on single species only, differed substantially in playback buzz rate and did usually not account for (baseline) conspecific activity. Our goal was to overcome these limitations and systematically test which inadvertent social information bats integrate when eavesdropping on others and how this integration affects space use and both intra‐ and interspecific interactions, respectively.
3. We used a community‐wide approach and investigated the effects of a broad range of playback feeding buzz rates and conspecific activity on eavesdropping responses in 24 bat species combinations in the wild.
4. For the first time, we reveal that finely graded and density‐dependent eavesdropping responses are not limited to particular foraging styles or call types, but instead are ubiquitous among insectivorous bats. All bats integrated social information about calling species identity, prey abundance and conspecific activity to estimate the cost–benefit ratio of prospective interactions, yet in a species‐specific manner. The effect of buzz rate was multifaceted, as bats responded differently to different buzz rates, and responses were additionally modulated by heterospecific recognition. Conspecific activity, in contrast, had a negative effect on the eavesdropping responses of all bats.
5. These findings can explain the inconsistent results of previous studies and advance our understanding of the complex nature of conspecific and heterospecific interactions within bat communities. A comprehensive understanding of how bats incorporate social information into their decision‐making will help researchers to explain species distribution patterns and eventually to unravel mechanisms of species coexistence.

## Graphical abstract

Social information improves decision‐making in animals. Here, we demonstrate that insectivorous bats ubiquitously eavesdrop on the echolocation calls of conspecific and heterospecific bats. All investigated species integrated social information about species identity, prey abundance and conspecific activity to estimate the cost–benefit ratio of prospective interactions, yet in a species‐specific manner.

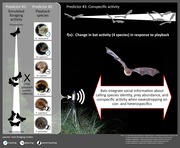

## Zusammenfassung

1. Tiere können Informationen nutzen, die von anderen Tiere unbeabsichtigt preisgegeben werden (sog. unbeabsichtigte soziale Informationen, engl.: *inadvertent social information*), um überlebenswichtige Entscheidungen zu treffen, z.B. darüber, wo es sich lohnt, nach Nahrung zu suchen oder mit wem es sich lohnt zu interagieren. Da Fledermäuse extrem laute, artspezifische Rufe aussenden, um im Flug zu navigieren und Beute zu jagen, bieten sie einen kontinuierlichen akustischen Strom an unbeabsichtigter sozialer Information an, der von anderen entschlüsselt werden kann. Besonders interessant für andere Fledermäuse sind die sog. ‚Fangrufe’ (engl.: *feeding buzz*) – charakteristische Ruffolgen, die jedem Beutefang vorausgehen und deren Häufigkeit mit der Insektenhäufigkeit korreliert.
2. Frühere Studien, die die Verwendung von unbeabsichtigter sozialer Information unter Fledermäusen untersucht haben, kamen zu unterschiedlichen, teils gegensätzlichen Ergebnissen. Diese Heterogenität ist vermutlich dadurch verursacht, dass diese Studien meist nur einzelne Arten betrachteten, sich erheblich in der wiedergegebenen Fangruf‐Häufigkeit unterschieden und die Ausgangs‐Aktivität der jeweiligen Fledermausart meist nicht berücksichtigten. Unser Ziel war es, diese Limitierungen zu überwinden und systematisch zu testen, welche unbeabsichtigten sozialen Informationen Fledermäuse integrieren, wenn sie die Rufe anderer Fledermäuse hören. Weiterhin wollten wir feststellen, wie diese Integration die Raumnutzung der Fledermäuse und inner‐ als auch zwischenartliche Interaktionen beeinflusst.
3. Dazu nutzten wir einen Forschungsansatz, der die gesamte Fledermaus‐Artengemeinschaft miteinbezieht. Im Freiland untersuchten wir den Einfluss einer großen Bandbreite von Fangruf‐Häufigkeiten und der Aktivität von Artgenossen auf die Reaktion von 24 Fledermausart‐Kombinationen.
4. Wir konnten zum ersten Mal zeigen, dass fein abgestimmte Verhaltensänderungen als Antwort auf die Rufe anderer Fledermäuse nicht auf einen bestimmten Jagd‐ oder Ruftyp beschränkt, sondern vielmehr weitverbreitet innerhalb der insektenfressenden Fledermäuse sind. Alle untersuchten Fledermäuse integrierten soziale Informationen über die Art der rufenden Fledermaus, über die Beutehäufigkeit und über die momentane Aktivität der Artgenossen in artspezifischer Art und Weise, um das Kosten‐Nutzen‐Verhältnis zukünftiger Interaktionen abzuschätzen. Der Effekt der widergegebenen Fangruf‐Häufigkeit war sehr divers, da Fledermäuse unterschiedlich auf verschiedene Fangruf‐Häufigkeiten reagierten und ihre Reaktionen zudem in Folge heterospezifischer Arterkennung angepasst haben. Die momentane Aktivität der Artgenossen hatte dagegen generell einen negativen Effekt auf das Lauschverhalten aller untersuchten Fledermausarten, so dass unsere Playbacks bei hoher natürlicher Aktivität der Artgenossen fast immer zu einer Abnahme der Aktivität führten.
5. Unsere Erkenntnisse können die uneinheitlichen Ergebnisse früherer Studien erklären und erweitern unser Verständnis der komplexen inner‐ und zwischenartlichen Interaktionen innerhalb von Fledermausgemeinschaften. Ein umfassendes Wissen darüber, wie Fledermäuse soziale Informationen in ihre Entscheidungen miteinbeziehen, wird dazu beitragen, die Verbreitungsmuster von Arten zu erklären und Mechanismen der Koexistenz von Arten zu entschlüsseln.

## 1 INTRODUCTION

All animals continuously have to make fitness‐relevant decisions, for example about engaging in social interactions or selecting breeding sites, mates and foraging grounds. To optimize decision‐making, individuals can acquire information themselves (personal information) or from other individuals (social information) before taking a decision. As opposed to intentional communication between sender and receiver(s) via signals, a universal means to acquire social information is eavesdropping, that is using cues that are inadvertently produced by others when being engaged in some activity such as foraging (Danchin, Giraldeau, Valone, & Wagner, 2004). Eavesdropping can be beneficial for decision‐making in a variety of ecological contexts (reviewed in Goodale, Beauchamp, Magrath, Nieh, & Ruxton, 2010; Valone & Templeton, 2002). Particularly when foraging on patchily distributed, ephemeral and thus unpredictable prey, inadvertent social information can aid localizing prey and reducing uncertainty about relative patch profitability, thus optimizing decisions on where to forage (Barta & Szép, 1992; Egert‐Berg et al., 2018; Gager, 2019; Valone & Templeton, 2002). Eavesdropping on conspecific cues has therefore evolved in a wide range of taxa, such as birds, fish and mammals (Gil, Hein, Spiegel, Baskett, & Sih, 2018). Yet, also eavesdropping on cues from sympatric heterospecifics can provide valuable information, since heterospecifics at the same trophic level often use similar resources and need to avoid similar predators (Goodale et al., 2010). While many aquatic organisms use olfactory or visual information, terrestrial animals often eavesdrop on acoustic cues (Coolen, Bergen, Day, & Laland, 2003; Goodale et al., 2010).

Echolocating bats are able to orientate and forage in three dimensions using acoustic information only by constantly emitting ultrasonic echolocation calls. Yet, ultrasonic frequencies attenuate quickly in air (Goerlitz, 2018). Therefore, bat echolocation call amplitudes are very high and reach 120–140 dB peSPL at 10‐cm distance to the mouth (Holderied & von Helversen, 2003), which corresponds to the pain threshold in humans (SCENIHR, 2008). Nevertheless, echoes returning from insects are comparably faint because of insects’ small size and corresponding small reflecting surface (Waters, Rydell, & Jones, 1995) and because echoes attenuate further when travelling back to the calling bat. In consequence, a bat will hear the echolocation calls of other bats over much larger distances than its own echoes, making echolocation calls an important informational resource prone to be eavesdropped upon (Jones & Siemers, 2011). Bat echolocation calls have evolved primarily under the selection pressure of orientation and foraging in darkness (Schnitzler, Moss, & Denzinger, 2003). Nevertheless, they can encode crucial information, such as calling species (Schuchmann & Siemers, 2010; Voigt‐Heucke, Taborsky, & Dechmann, 2010), sex (Kazial & Masters, 2004; Schuchmann, Puechmaille, & Siemers, 2012), social affiliation (group, colony; Voigt‐Heucke et al., 2010) and even individual identity (Voigt‐Heucke et al., 2010; Yovel, Melcon, Franz, Denzinger, & Schnitzler, 2009). Thus, bats may use both social and echolocation calls for social communication and decision‐making (Fenton, 2003; Jones & Siemers, 2011). Yet only echolocation calls are constantly emitted at high rate when bats are flying.

Once a bat detects flying prey and initiates the attack, the bat progressively increases call rate and bandwidth, culminating in a stereotypical sequence of superfast calls (up to 210 Hz; Kalko & Schnitzler, 1989) right before capture, the terminal feeding buzz (short: buzz). Since every prey capture is preceded by a buzz, the rate of feeding buzzes correlates strongly with insect density (Racey & Swift, 1985) and thus might be of special interest for other bats (Gager, 2019). Especially, insects in riparian habitats, such as swarms of emerging Chironomidae, are often patchily distributed in space, hardly predictable in time, and usually too numerous to be monopolized (Jones & Rydell, 2003). Hence, individuals of the trawling lesser bulldog bat *Noctilio albiventris* stay in hearing distance to each other for most of the time (Dechmann et al., 2009) and likely eavesdrop on conspecifics’ feeding buzzes to localize insect aggregations. Group foraging only becomes detrimental at very high bat densities since bats then have to focus their attention on close‐by conspecifics to avoid physical collision and cannot forage at the same time (Cvikel et al., 2015). However, not all trawling bats necessarily perform group hunting; the Daubenton's bat *Myotis daubentonii*, for instance, seems to defend small foraging patches and chases intruders away (Encarnacao, Becker, & Ekschmitt, 2010). Similarly, aerial‐hawking common pipistrelle bats *Pipistrellus pipistrellus* defend foraging patches against conspecifics when prey availability is low (Barlow & Jones, 1997; Racey & Swift, 1985).

To date, several bat species have been shown to eavesdrop on search calls and feeding buzzes of other bats, yet responses vary substantially (Balcombe & Fenton, 1988; Barclay, 1982; Dechmann et al., 2009; Dorado‐Correa, Goerlitz, & Siemers, 2013; Egert‐Berg et al., 2018; Gillam, 2007; Hügel et al., 2017; Jonker, Boer, Kurvers, & Dekker, 2010; Li et al., 2014; Roeleke, Johannsen, & Voigt, 2018; Übernickel, Tschapka, & Kalko, 2013). Most studied species seem to be attracted towards conspecific feeding buzzes, but in some cases, they are not attracted or are even repelled (reviewed in Gager, 2019). Literature on responses towards heterospecific feeding buzzes is scarce, and responses vary even more. Strikingly, studies empirically testing eavesdropping on feeding buzzes have used a wide range of playback buzz rates from 1 to >60 buzzes per minute. Additionally, the actual bat activity prior to playbacks has rarely been taken into account, although it strongly varies over time and space and can considerably affect eavesdropping in bats (Roeleke et al., 2018). Together, these differences between studies might explain a large part of the observed variability in bats’ responses between studies and complicate drawing general conclusions.

In this study, we aimed to reduce this gap of knowledge by systematically testing for an effect of feeding buzz rate on eavesdropping behaviour in a free‐ranging bat community while at the same time controlling for actual bat activity. We hypothesized that eavesdropping in bats mainly depends on an individual's expected cost–benefit ratio, that is that bats would respond differently to low and high feeding buzz rates of different species. When bats eavesdrop on other bats to identify rich foraging patches, we predicted increasing positive phonotaxis both with increasing dietary overlap and with increasing buzz rate. In contrast, when bats eavesdrop on others to defend foraging patches against intruders, we similarly predicted positive phonotaxis to increase with dietary overlap but not with feeding buzz rate. For both situations, we predicted that positive phonotaxis would decrease with increasing bat density due to sensorial constraints and increased competition. To test our predictions, we conducted a large‐scale field experiment at 12 central European lakes, where we broadcast a wide range of feeding buzz rates to the local bat community. We used playbacks from six bat species that differed in dietary overlap, foraging habitats, foraging strategy and call structure. This experimental design enabled us to investigate systematically the influence of the expected cost–benefit ratio on eavesdropping propensity on both conspecifics and heterospecifics, while at the same time shedding light on which cues bats use to optimize their decision‐making.

## 2 MATERIALS AND METHODS

### 2.1 Playback generation

Our pool of bat call sequences contained 744 short (1.25 s) search phase and feeding buzz sequences of long‐fingered bats *Myotis capaccinii*, Daubenton's bats *Myotis daubentonii*, Natterer's bats *Myotis nattereri*, Leisler's bats *Nyctalus leisleri*, common pipistrelles *Pipistrellus pipistrellus* and soprano pipistrelles *Pipistrellus pygmaeus* (56, 23, 17, 13, 55 and 22 sequences, respectively, for both search and buzz sequences). These playback species covered different call types (from short *Myotis* to long *Nyctalus* calls) and habitat preferences (open water, clutter, open air) and in consequence differed concerning prey species overlap. Thus, choice of these playback species allowed for testing the “acoustic similarity hypothesis” and the “foraging similarity hypothesis” as described in detail in Hügel et al. (2017) and to directly compare our results with results from that study. In addition to the five playback species of Hügel et al. (2017), we included common pipistrelle playback because this species is very abundant at our study sites and its foraging habitat is intermediate between the open space used by Leisler's bats and the cluttered riparian habitats used by soprano pipistrelles.

We normalized the peak amplitude of the loudest call of each sequence to 90% full scale. To generate 1‐min stereo playback files, we randomly picked 96 sequences of the same species, with either 0, 12, 24, 48 or all 96 being feeding buzz sequences (and the remaining ones being search call sequences). Buzz sequences were assigned pseudo‐randomly (Gellermann, 1933) to either of the two stereo channels. Each channel thus contained *n* = buzz rate/2 buzz sequences embedded in consecutive search call sequences (i.e. in total 48 1.25‐s‐long sequences per channel; Figure S1). Each channel was compensated for the impulse response of the speaker that it was going to be broadcast with and high pass filtered at 10 kHz (8th‐order butterworth). We prepared new playback files for every experimental night, such that each unique playback file was only used once. As control, we played an empty file of 1‐min duration both before and after each 1‐min playback file. Each playback block thus lasted 3 min and comprised three phases: pre‐playback, playback and post‐playback phases (Figure S1).

### 2.2 Playback broadcast and sound recordings

We conducted the playback experiment at 12 different lakes in Southern Germany (Figure S2) during 12 evenings without precipitation in July and August 2016. At each site, we broadcast each playback species—buzz rate combination only once in random order using Recorder Software, UltraSoundGate Player 216H, and two Vifa speakers (all Avisoft Bioacoustics), resulting in 90 min (six species × five buzz rates × 3 min each) of playback (Figure S1). Playback volume was maximized without clipping, resulting in maximum playback amplitudes of 115 ± 2 dB SPL at 10 cm (mean ± *SD*). Assuming a bat hearing threshold of 20 dB, low playback frequencies (20 kHz) were audible over 89 m while higher frequencies (50 kHz) that suffer stronger atmospheric attenuation reached over 27 m at 20°C and 70% relative humidity. While broadcasting playback, we continuously recorded bat calls with an omni‐directional Knowles FG‐O microphone and UltraSoundGate 416H soundcard (300 kHz sample rate, 30 dB gain; Avisoft Bioacoustics) as WAV files. The microphone was positioned 1.2 ± 0.4 m (mean ± *SD*) off the shore at 1.7 ± 0.2 m and 2.1 ± 0.3 m above‐ground and water level, respectively, pointing towards the lake (perpendicular to the shoreline). The two speakers were placed at the shoreline 0.8 ± 0.4 m above water level and 3.2 ± 0.7 m right and left of the microphone, pointing about 45° to the left and right off the microphone direction, respectively. We started playback and recording at 42 ± 8 min after sunset.

### 2.3 Sound analysis

Recordings were analysed manually in SASLab Pro software (Avisoft Bioacoustics) blind to experimental phase and playback species. We only considered calls with a recorded amplitude above −60 dB FS. For each second recorded, we determined the species present and the number of individuals per species based on the frequency–time structure of the calls and temporal patterns of call sequences (Figure S3). Yet, due to largely overlapping frequency–time call structures and parameters of call sequences, exclusively acoustic identification to species level is unreliable for most *Myotis* species and often also for Leisler's bats, noctule bats *Nyctalus noctula*, northern bats *Eptesicus nilssonii*, serotine bats *E. serotinus* and particoloured bats *Vespertilio murinus*. Fortunately, however, the Daubenton's bat was most likely the only *Myotis* species at the lake study sites where the Daubenton's bat was the only trawling bat species. We could visually confirm the presence of Daubenton's bats in proximity to the microphones in most cases based on the species’ typical flight just above the water surface. Thus, we treated all *Myotis* calls as the Daubenton's bat. We grouped all bats of the genera *Nyctalus*,* Eptesicus* and *Vespertilio* as species group “NEV,” which is common practice (e.g. Lewanzik & Voigt, 2017) and mirrors to some extent these species’ ecology as narrow‐winged fast‐flying open‐space foragers. At the study sites, however, the vast majority of NEV calls are likely from noctule bats; about a third of all NEV group bat‐seconds (see below) could be unambiguously attributed to this species.

### 2.4 Statistical analysis

We measured species‐specific activity as the number of seconds per minute of playback in which a species (or species group) was identified. If multiple individuals of the same species (group) were identified in the same second, that second was counted multiple times accordingly (Figure S3). Consequently, activity was measured as bat‐seconds per minute and could be larger than 60. To construct our response variable *activity change*, we subtracted the conspecific activity measured in the minute before any given playback minute (i.e. without bat calls being broadcast; hereafter short “conspecific activity”) from the activity in minutes with playback. We excluded 3‐min playback blocks from our analyses in which we did not detect any call of a given response species in both pre‐playback and playback phases. Furthermore, we did not analyse activity of soprano pipistrelles and unidentified bats since both accounted for <1% of overall bat‐seconds. In total, we analysed 250, 94, 110 and 180 playback blocks for Daubenton's bats, NEV group bats, Nathusius’ pipistrelles and common pipistrelles, respectively (Table S1).

Each response species (group) was analysed separately using the statistical freeware r version 3.4.4 (R Core Team, 2018). We used the *lmer* function of the R‐package *lme4* (Bates, Maechler, Bolker, & Walker, 2015) to fit linear mixed‐effects models with a Gaussian error distribution and *activity change* as response variable. *Activity change* was modelled as a function of the fixed effects *buzz rate* (continuous), *squared buzz rate* (continuous), *playback species* (categorical with six levels), *conspecific activity* (continuous), *presence of other species* (continuous dummy variable coded as “0” or “1”) and *minutes after sunset* (continuous). We included a quadratic buzz rate term because the effect of feeding buzz rate was predicted to be nonlinear (Jonker et al., 2010). We included the fixed effect *presence of other species* since the presence of other real bats likely influences bats’ responses towards the simulated (playback) bats. Interaction terms included in the models were *buzz rate* × *playback species* and *squared buzz rate* × *playback species*. All continuous predictors were standardized (i.e. centred on 24 buzzes/min (buzz rate), on mean conspecific activity or on 60 min after sunset, respectively, and scaled by their *SD*). To meet assumptions of the Weber–Fechner law of perception, we transformed buzz rates as log2(buzz rate/12) + 2 and conservatively set log2(0/12) + 2 to 1, such that the buzz rates we broadcast (0, 12, 24, 48, 96 buzzes/min) were transformed to equally spaced values (1, 2, 3, 4, 5). For each of the four response species (groups), we defined either conspecific playback as reference playback species (Daubenton's bats, common pipistrelles) or the playback species with the most similar foraging ecology (for NEV group: Leisler's bat; for Nathusius’ pipistrelle: soprano pipistrelle). Model intercepts were omitted, and in consequence, estimates for the six levels of *playback species* were compared with zero instead of with the reference level estimate. To account for the dependency among observations at the same site, we included *site* as random effect in the models (categorical with 12 levels). Model assumptions were verified by plotting residuals versus fitted values and by inspecting QQ‐plots of the model residuals and of the random effect. We assessed residuals for temporal dependency by plotting estimates of the autocorrelation function using R's acf function.

We used the *sim* function of the r‐package *arm* (Gelman & Su, 2016) to simulate the values of the posterior distributions of the model parameters (1,000 simulations) and to obtain posterior mean parameter estimates. We extracted 95% credible intervals (CrIs) around the posterior mean estimates and considered support for an effect as strong if zero was not included within the 95% CrI and as low if overlap with zero was small, respectively. If 95% CrIs were centred on zero, we considered this as strong support for the absence of an effect. We also used the *sim* function to obtain mean predictions of activity change and 95% CrIs around these predictions for varying buzz rates, playback species and baseline conspecific activities while fixing *minutes after sunset* to 60 (i.e. 1 hr after sunset) and *other species* to 0 (i.e. no other species present).

## 3 RESULTS

The Daubenton's bat was the most common species at our recording sites, comprising 50% of all bat activity. Nathusius’ pipistrelles, common pipistrelles and bats in the NEV group accounted for 23%, 14% and 13% of bat activity, respectively. Besides the Nathusius’ pipistrelle, which was not recorded at one lake, all other species (groups) were identified at all 12 lakes, yet variation in bat‐seconds among lakes was large (median [min‐max]; Daubenton's bat: 649 [168–2,776]; Nathusius’ pipistrelle: 153 [0–2,815]; common pipistrelle: 271 [55–493]; NEV: 69 [8–1,242]). Results are presented for no (0 bat‐seconds/min), mean and maximum conspecific activity (as measured during the pre‐playback minute), of which the latter two are species‐specific: 15, 11, 18, 7 bat‐seconds/min (mean) and 98, 61, 81, 56 bat‐seconds/min (max) for Daubenton's bats, NEV group, Nathusius’ pipistrelles and common pipistrelles, respectively.

**Figure 1:**
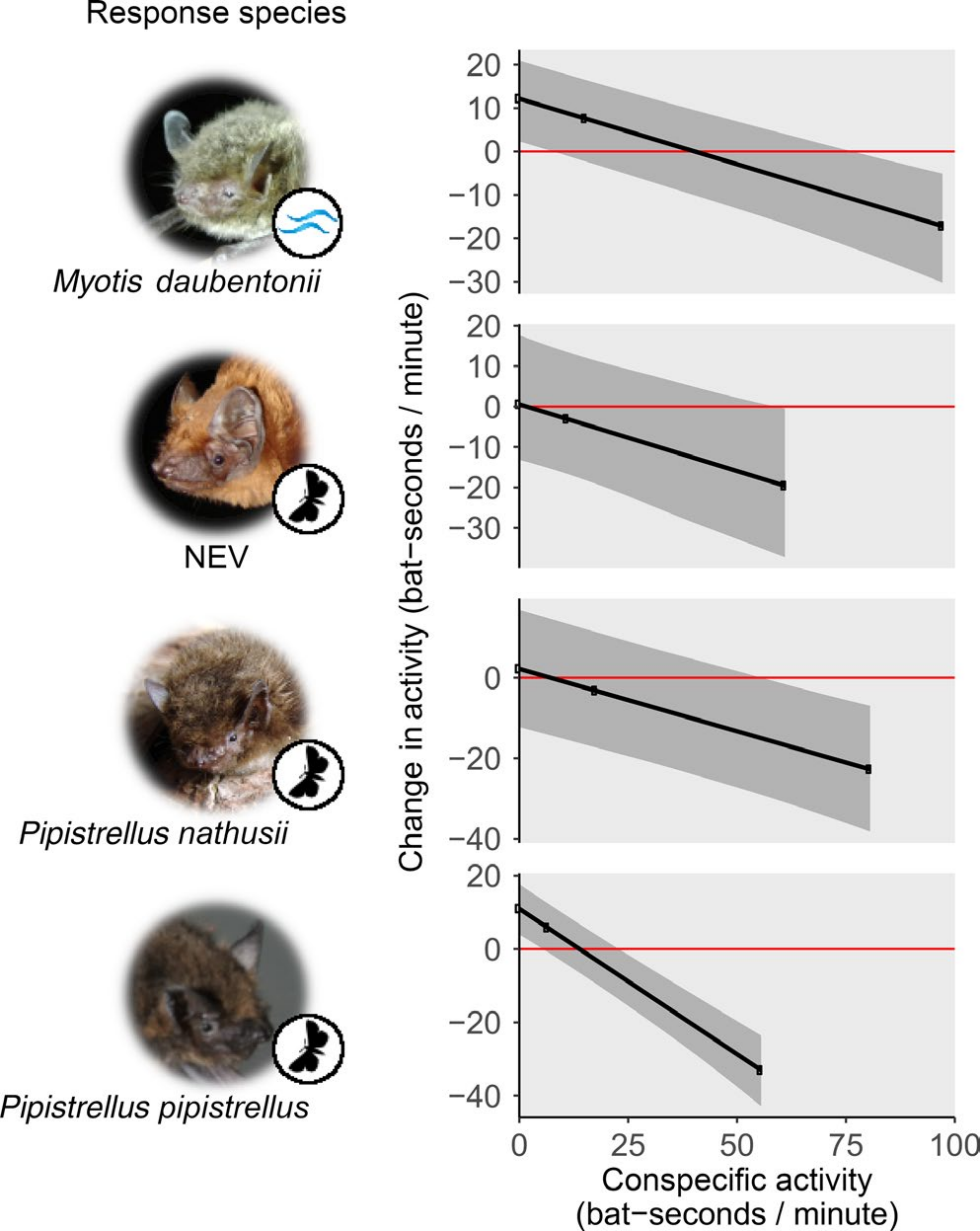
Exemplary effect of conspecific activity on activity changes in all four response species (groups), for playback of reference species search call sequences (i.e. buzz rate = 0) at 1 hr after sunset without other species present. Values above and below zero (red line) indicate activity increases and decreases, respectively. Without any conspecific activity, our playback of bat echolocation sequences increased bat activity or had no effects, while it reduced bat activity at maximum conspecific activity. Predictions and 95% CrIs are depicted as black lines and grey shaded areas, respectively. Predictions at no, mean, and maximum conspecific activity (measured in pre‐playback minute) are highlighted by small black dots. Symbols at pictures indicate main foraging modes: waves = trawling, moth = aerial‐hawking. Please note species‐specific y‐scales. Photo copyrights as in Figure S4

**Figure 2:**
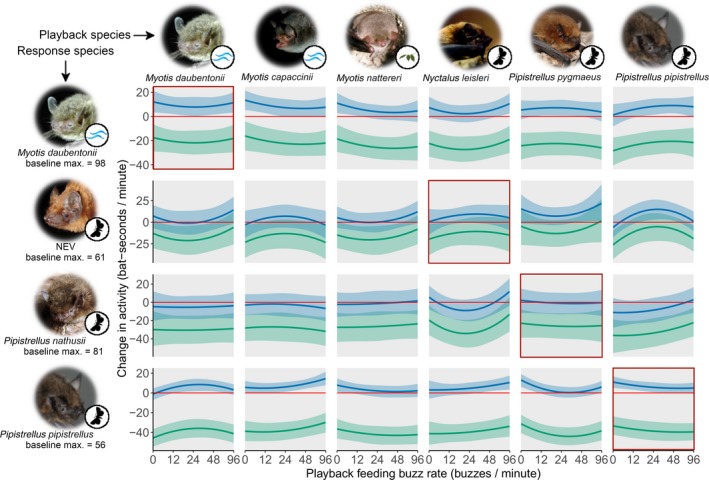
Change in bat activity between control (silence) and treatment (search/buzz call playback) minutes for 24 combinations of playback and response species. Baseline conspecific activity is the actual bat activity in the control minute preceding a given treatment minute and was measured during data analysis (blue: no conspecific activity, i.e. 0 bat‐seconds/min; green: species‐specific maximum conspecific activity as indicated below pictures). One‐minute playbacks contained feeding buzzes embedded in search call sequences at rates of 0, 12, 24, 48 or 96 buzzes/min. Predictions and 95% CrIs (solid curved lines and shaded areas, respectively) are calculated for 1 hr after sunset, assuming no other species were present. Values above and below zero (horizontal red line) indicate activity increases and decreases, respectively. Red boxes highlight responses towards reference species. Symbols at pictures indicate main foraging modes: waves = trawling, leaves = gleaning, moth = aerial‐hawking. Please note logarithmic x‐scale and species‐specific y‐scales. Photo copyrights as in Figure S4

While playback feeding buzz rate had species‐specific effects on bat activity changes (see below), conspecific activity was strongly negatively correlated with the activity changes in all four response species (Figure 1, Table 1). At both no conspecific activity and mean conspecific activity, activity changes were almost all positive (i.e. activity increased) or neutral (see Figure 1 for exemplary effects per response species; Figure 2 and Figure S4 for effects per response/playback species and buzz rate). At maximum conspecific activity, in contrast, activity of all response species almost exclusively decreased when broadcasting bat calls (Figures 1 and 2). Bats of the NEV group constitute an exception, however, as they reduced activity only in response to some but not all buzz rates when conspecific activity was at its maximum (Figure 2).

**Table 1:** Posterior mean estimates and 95% credible intervals (CrIs) for fixed effects and interactions in the models of bat activity changes for each response species (group). Effects for which CrI does not overlap with zero are indicated by asterisks

Fixed effect or interaction	*Myotis daubentonii*			NEV			*Pipistrellus nathusii*			*Pipistrellus pipistrellus*		
	Estimate	2.5% CrI	97.5% CrI	Estimate	2.5% CrI	97.5% CrI	Estimate	2.5% CrI	97.5% CrI	Estimate	2.5% CrI	97.5% CrI
Buzz rate	−0.25	−4.02	3.90	1.55	−4.90	8.05	−0.93	−5.99	4.59	−2.16	−4.86	0.57
(buzz rate)^2^	1.94	−3.13	6.26	−3.04	−9.46	3.59	0.98	−4.42	6.76	1.16	−2.14	4.43
*M. cappaccinii* playback	2.67	−4.03	9.59	3.39	−11.41	16.94	−7.57	−20.86	5.28	0.88	−4.38	5.81
*M. daubentonii* playback	3.21	−3.24	10.35	−4.48	−18.52	9.02	−10.80	−22.75	1.09	3.16	−2.93	8.83
*M. nattereri* playback	−0.68	−6.91	5.42	−3.54	−16.25	8.47	−6.85	−19.40	7.26	−3.16	−8.98	2.66
*N. leisleri* playback	−2.23	−9.68	5.36	5.64	−5.76	16.32	−14.35	−26.18	−1.27*	−0.54	−6.04	5.52
*P. pipistrellus* playback	3.69	−3.42	10.25	11.17	−0.57	22.49	−14.03	−25.47	−0.47*	0.49	−4.76	5.18
*P. pygmaeus* playback	2.54	−3.49	9.21	3.67	−7.77	14.75	−6.56	−18.40	5.07	−4.51	−9.51	1.02
Conspecific activity	−5.66	−7.52	−3.95*	−4.54	−7.06	−1.70*	−5.93	−8.41	−3.50*	−6.34	−7.64	−5.07*
Presence of other species	1.38	−1.89	4.73	−1.28	−8.20	6.80	7.67	−2.04	16.68	0.45	−2.43	3.46
Minutes after sunset	−1.06	−3.43	1.60	1.43	−2.23	5.63	0.43	−2.45	3.70	−1.03	−2.93	0.73
Buzz rate : *M. cappaccinii* playback	−1.79	−6.95	3.66	−1.65	−11.35	8.22	−0.38	−8.15	6.68	5.32	1.21	9.63*
Buzz rate : *M. daubentonii* playback	—	—	—	0.85	−8.38	11.05	1.28	−6.47	9.12	3.69	0.10	8.02*
Buzz rate : *M. nattereri* playback	−1.99	−7.68	3.64	0.77	−7.84	9.17	2.27	−5.93	9.65	0.12	−4.09	4.43
Buzz rate : *N. leisleri* playback	1.38	−4.44	6.38	—	—	—	3.15	−4.54	10.52	4.84	0.09	9.08*
Buzz rate : *P. pipistrellus* playback	2.61	−3.04	8.42	0.89	−7.89	9.85	5.82	−2.05	13.86	—	—	—
Buzz rate : *P. pygmaeus* playback	−0.33	−5.86	4.84	0.64	−9.93	10.78	—	—	—	−0.37	−4.50	3.39
(buzz rate)^2^: *M. cappaccinii* playback	−0.35	−6.88	6.36	−1.76	−12.42	8.39	−2.34	−10.25	5.96	0.86	−3.66	5.64
(buzz rate)^2^: *M. daubentonii* playback	—	—	—	8.52	−1.46	18.77	−0.43	−9.07	8.22	−4.96	−9.72	−0.29*
(buzz rate)^2^: *M. nattereri* playback	−0.04	−6.35	6.32	7.12	−2.67	16.67	−0.46	−9.42	7.83	0.36	−5.10	5.45
(buzz rate)^2^: *N. leisleri* playback	1.25	−5.26	7.81	—	—	—	7.72	−1.44	16.50	−0.05	−5.48	4.95
(buzz rate)^2^: *P. pipistrellus* playback	−3.73	−10.01	2.99	−5.11	−14.42	4.12	1.21	−8.12	9.11	—	—	—
(buzz rate)^2^: *P. pygmaeus* playback	−3.21	−9.31	2.94	8.44	−2.32	19.83	—	—	—	3.37	−1.24	7.92

Below we present detailed descriptions of the responses towards different feeding buzz rates and varying conspecific activity for each response species separately. Posterior mean predictions of activity change are calculated for 1 hr after sunset assuming no other species were present. None of these two predictors, “time after sunset” and “presence of other species,” affected activity changes in any response species (Table 1).

### 3.1 The Daubenton's bat

Without any conspecific activity, Daubenton's bats increased activity in response to every playback species, with different effects of buzz rate depending on playback species. When broadcasting calls of trawling *Myotis* species (i.e. conspecifics and long‐fingered bats), Daubenton's bats increased activity over all feeding buzz rates presented, with highest activity increases at very low buzz rates (Figure 2). When broadcasting calls of gleaning Natterer's bats, Daubenton's bats increased activity only with low playback feeding buzz rates (Figure 2). When broadcasting non‐*Myotis* species, in contrast, Daubenton's bats increased activity mainly at intermediate or high feeding buzz rates (Figure 2).

At mean conspecific activity, Daubenton's bats increased activity only in response to very low feeding buzz rates of *Myotis* species (strong support for long‐fingered bat playback, less support for Daubenton's and Natterer's bat playback), but not in response to any buzz rates of non‐*Myotis* species (Figure S4, Table 1).

When conspecific activity was maximal, Daubenton's bat activity decreased in response to all playback species and feeding buzz rates (Figure 2, Table 1).

### 3.2 NEV group bats

Without any conspecific activity, NEV group bats increased activity mainly in response to conspecific and heterospecific aerial‐hawking bats; activity increased in response to high soprano pipistrelle and intermediate common pipistrelle buzz rates and tended to increase at intermediate Leisler's bat and low soprano pipistrelle buzz rates, too (Figure 2). In response to playback of trawling Daubenton's and gleaning Natterer's bats, NEV group bats tended to increase activity only at very high playback buzz rates (Figure 2).

At mean conspecific activity, NEV group bats were unresponsive to almost all playback species and feeding buzz rates; only intermediate buzz rates of common pipistrelles tended to cause an increase in their activity (Figure S4, Table 1).

When conspecific activity was maximal, NEV group bats decreased activity in response to all playback species but—in contrast to the other three response species—not over all playback buzz rates. Particularly, at high Daubenton's bat buzz rates, low and high soprano pipistrelle buzz rates and intermediate common pipistrelle buzz rates NEV group bats did not change activity (Figure 2). Also, at certain buzz rates of other playback species support for an activity decrease was only low since 95% CrIs overlap slightly with zero (Figure 2, Table 1).

### 3.3 The Nathusius’ pipistrelle

Without any conspecific activity, we did not find strong support for any activity changes in Nathusius’ pipistrelles. Only in response to two aerial‐hawking playback species, Nathusius’ pipistrelles tended to change activity; that is, they decreased activity at intermediate Leisler's bat and low common pipistrelle buzz rates, but increased activity at high Leisler's bat buzz rates (Figure 2).

At mean conspecific activity, Nathusius’ pipistrelles decreased activity in response to intermediate Leisler's bat and low common pipistrelle buzz rates and also tended to decrease activity in response to all Daubenton's bat buzz rates (Figure S4, Table 1).

When conspecific activity was maximal, Nathusius’ pipistrelles decreased activity in response to every playback species and feeding buzz rate (Figure 2, Table 1).

### 3.4 The common pipistrelle

Without any conspecific activity, common pipistrelles increased activity in response to every playback species. Activity increases were most pronounced either at low buzz rates (conspecific, soprano pipistrelle and Natterer's bat playback), at intermediate buzz rates (Daubenton's bat playback) or at high buzz rates (Leisler's and long‐fingered bat playback; Figure 2).

At mean conspecific activity, common pipistrelles responded to playbacks with both activity increases and decreases (Figure S4). They increased activity in response to low congeneric (i.e. soprano pipistrelle) buzz rates and tended to do so in response to conspecific playback as well. Activity increases were even more pronounced in response to high buzz rates of long‐fingered bats. They decreased activity when broadcasting low buzz rates of Daubenton's bats and tended to decrease activity during intermediate buzz rates of soprano pipistrelles.

When conspecific activity was maximal, however, common pipistrelles decreased activity in response to all playback species and across all playback feeding buzz rates (Figure 2, Table 1).

## 4 DISCUSSION

Our results illustrate the diversity and complexity of interspecific interactions in bat communities, highlighting that inferences from single species studies are not sufficient to understand population dynamics and space use of bats. For the first time, we experimentally tested the use of inadvertent social information among 24 combinations of bat species in the wild. We demonstrate that the investigated bat species all make use of social information as the (simulated) presence and foraging activity of other individuals affected their behaviour. Yet, the degree to which species used available information, the way they responded to it, and the species they eavesdropped upon substantially differed between species. These differences between species strongly support the hypothesis that the strength of heterospecific interactions is modulated by heterospecific recognition. Most importantly, however, we also demonstrate that responses of all four investigated species (groups) towards the calls of other bats depended on the feeding buzz rate presented, particularly when conspecific activity was low. Similarly, conspecific activity had a strong effect on the responses of all response species and even inverted activity changes from an increase at low conspecific activity to a decrease at high conspecific activity. Thanks to our community‐wide approach, we reveal for the first time that finely graded and density‐dependent eavesdropping responses are not limited to particular foraging styles or call types but instead are ubiquitous among insectivorous bats.

Previous work aimed at testing the “acoustic similarity hypothesis” and the “foraging similarity hypothesis”, which assume that bats are generally attracted to calls of other bats if the acoustic call structure or if the foraging ecology is similar to their own, respectively (Balcombe & Fenton, 1988; Hügel et al., 2017; Übernickel et al., 2013). While these hypotheses might be useful to explain differences in eavesdropping responses within some bat species, our results suggest that neither of them is sufficient to explain the diverse responses across different bat species. We propose instead that bats generally trade‐off costs and benefits of possible responses towards other bats and, in consequence, responses depend on a multitude of factors such as a bats’ behavioural context (e.g. foraging, territory defence, mating), prey abundance, conspecific and heterospecific densities, acoustic call parameters, preferred foraging habitat and competitive abilities, among others. In the following, we discuss our results according to our predictions, that is separately for bats that eavesdrop on others to localize good foraging opportunities and for those that defend feeding patches and eavesdrop on others to spot intruders. As we predicted the effect of conspecific activity to be independent of behavioural context, we discuss this effect jointly for all bats.

### 4.1 Eavesdropping to find and judge foraging opportunities

When eavesdropping on others to improve foraging efficiency, not only the number and density of prey items, but also their accessibility determines the cost–benefit ratio for the eavesdropping bat. Small insects might be more difficult to detect by the low‐frequency echolocation of larger bats (Pye, 1993; but see Waters et al., 1995), while small bats might have difficulties handling larger prey. Furthermore, the long‐range echolocation and fast flight of open‐space aerial‐hawking bats are not suited to detect and prey on non‐flying arthropods which gleaning species readily take from vegetation. As predicted, dietary overlap affected the eavesdropping responses as none of the response species reacted in the same way to all playback species. This playback species specificity corroborates that bats distinguish to some extent between various heterospecifics (“heterospecific recognition”) and highlights that particular traits of the playback species affected the response species’ propensity to react to it. However, we could not identify a general response pattern; trawling Daubenton's bats as well as aerial‐hawking NEV group bats and common pipistrelles increased activity in response to feeding buzz playback from species of the same but also of other foraging guilds. It is thus likely that explanations for the observed response patterns are specific to the particular combination of playback species and response species. For instance, dietary overlap of the common pipistrelle is large with both the Daubenton's bat and the soprano pipistrelle, both of which forage mainly on aquatic insects (Diptera). Although common pipistrelles and soprano pipistrelles (but not Daubenton's bats) belong to the same guild of aerial hawkers, common pipistrelles increased activity in response to intermediate playback buzz rates only of Daubenton's bats. This finding might be a consequence of common pipistrelles not being able to compete with soprano pipistrelles, since the latter commonly forage in more cluttered habitats than common pipistrelles (Nicholls & Racey, 2006). Likely, it is difficult for common pipistrelles to move from their normal to a more cluttered habitat (Schnitzler et al., 2003). Common pipistrelles actively avoid soprano pipistrelle home ranges and rather fly to more distant and lower quality foraging sites than to share a foraging patch with soprano pipistrelles (Nicholls & Racey, 2006). Thus, our prediction of increasing positive phonotaxis with increasing dietary overlap seems to be generally true, but it is constrained by the level of clutter a bat has to deal with when foraging in the habitat of another species.

As opposed to all other response species, our results did not provide strong support for any activity changes in Nathusius’ pipistrelles in response to any playback species. This reluctance is in line with previous studies showing that Nathusius’ pipistrelles increase activity only in response to conspecific playback (which we did not present in our study) but not to heterospecific calls (Dorado‐Correa et al., 2013; Marggraf et al. (in review) in Roeleke et al., 2018). In contrast, Roeleke et al. (2018) showed recently that the noctule bat, which accounts for the vast majority of calls assigned to the NEV group in our study, responded more strongly to heterospecific Nathusius’ pipistrelle than to conspecific feeding buzzes. The authors argue that intraspecific competition might be stronger than interspecific competition due to the occupation of the same foraging space and echolocation call frequency range; given that prey is not a limiting factor. Similarly, we found that NEV group bats reacted more strongly towards the two pipistrelle playback species than to their reference species (i.e. Leisler's bat), which is in contrast to our prediction that positive phonotaxis increases with dietary overlap. However, at our experimental sites, NEV group activity was least affected by high conspecific activity compared with the other species investigated, suggesting that conspecifics are less likely to cause interference in the open air than in more spatially restricted habitats, as used by *Pipistrellus* species, for instance. Thus, we think that NEV bats increased activity more in response to pipistrelles than to Leisler's bats due to differences in foraging strategy between these species. Both noctule and Leisler's bats do usually not forage within defined foraging patches but instead over large areas in very fast and rather straight flight, thereby rather randomly encountering individual prey (Dietz, von Helversen, & Nill, 2007). Common and soprano pipistrelles, in contrast, often forage for extended times on small spatial scale on dense insect aggregations (Davidson‐Watts & Jones, 2006; Dietz et al., 2007; Racey & Swift, 1985). Thus, buzzes from these pipistrelles are more likely to indicate a sharable resource than buzzes from Leisler's bats, particularly as prey overlap between noctule bats and pipistrelles can be substantial despite size and foraging style differences (Arlettaz, Godat, & Meyer, 2000; Jones, 1995; Sullivan, Shiel, McAney, & Fairley, 1993). Accordingly, these results suggest that eavesdropping pays off when foraging on spatially or temporally unpredictable but dense prey clusters but less so when foraging on uniformly distributed low‐density prey.

As buzz rates correlate tightly with insect density (Racey & Swift, 1985), high feeding buzz rates indicate a large number of potential prey insects. In contrast, low buzz rates (i.e. mainly search calls) only announce the presence of another bat but not of plentiful prey. As predicted, we found that without any conspecific activity, bats of the NEV group increased activity more in response to intermediate and high than to low feeding buzz rates. These buzz rate‐dependant changes in activity suggest that NEV group bats use inadvertent social information to estimate relative prey numbers within prey clusters. One interesting exception that contrasts our prediction is the positive response of NEV group bats not only towards high but also towards low buzz rates of soprano pipistrelles when conspecific activity was zero. Soprano pipistrelles commute to fewer and further distant but therefore higher quality foraging sites than common pipistrelles, for instance (Davidson‐Watts, Walls, & Jones, 2006; Nicholls & Racey, 2006). Thus, we speculate that NEV group bats might benefit from following commuting soprano pipistrelles to their rich foraging grounds and are thus attracted not only to high feeding buzz rates but also to search calls of soprano pipistrelles.

### 4.2 Eavesdropping to spot intruders

Eavesdropping on heterospecific calls to obtain knowledge about the calling species is valuable not only for bats searching for prey, but also for those bats defending a foraging patch. If the heterospecific bat does not feed on the prey that is abundant in my patch, it will not be economic to invest energy into chasing the heterospecific off. Both Daubenton's bats and common pipistrelles exhibit temporary territoriality and chase conspecific intruders off their foraging patch when food is scarce (Encarnacao et al., 2010; Racey & Swift, 1985). Thus, we argue that the activity increases in these species in response to very low buzz rates of conspecifics and congenerics mirror the attempt of resident bats to defend their foraging patch against (simulated) intruders. As predicted, this was mainly the case for conspecifics and congenerics that overlap substantially in diet. Surprisingly, however, both Daubenton's bats and common pipistrelles increased activity also when broadcasting search calls of Natterer's bats, which are commonly regarded as a gleaning species. However, the foraging mode of Natterer's bats is flexible and can switch between gleaning and aerial‐hawking (Dietz et al., 2007). Given that Natterer's bats might compete with common pipistrelles for the same resources when aerial‐hawking, the observed increase in common pipistrelle activity in response to Natterer's search calls is in line with our prediction that positive phonotaxis would be dependent on dietary overlap. For Daubenton's bats, we attribute this supposedly antagonistic behaviour towards bats of another feeding guild to the high similarity in call shape among *Myotis* species, making it likely difficult to correctly identify the conspecific. This is confirmed by the increased activity in response to low buzz rates of any *Myotis* species, including even low buzz rates of long‐fingered bats, which are absent from our study sites and thus unknown to the bats we studied here.

In contrast to our prediction that bats defending a feeding patch would not differentiate between broadcast buzz rates, positive phonotaxis of Daubenton's bats and common pipistrelles generally decreased with increasing buzz rates of conspecifics and congenerics. We assume that the high buzz rates we broadcast (up to 96 buzzes/min) did not only indicate large insect numbers but also multiple bat individuals since single bats emit buzzes at much lower rate [*E. nilssonii*: 2–3/min (Rydell, 1992); *M. capaccinii/daubentonii*: 1/min (Hügel et al., 2017); *P. pipistrellus*: <10/min (Racey & Swift, 1985), 14/min (Kalko, 1995)]. The likelihood of successfully chasing multiple bats off at the same time is probably low, and motivation to do so accordingly, which is likely why activity increases were diminished in response to high buzz rates.

### 4.3 Conspecific activity affects eavesdropping responses of all bats

On top of the different effects of buzz rate and playback species, all the species we investigated adjusted their eavesdropping behaviour in a density‐dependent manner, highlighting the complexity of intra‐ and interspecific interactions. The many factors influencing eavesdropping complicate comparing field studies which did not account for them. For instance, previous studies suggest that trawling bats, such as Daubenton's bats, only approach feeding buzzes of conspecifics and of other trawling species, but not of non‐trawling bats (Dorado‐Correa et al., 2013; Hügel et al., 2017). While our findings at mean conspecific activity support this conclusion, Daubenton's bats also approached open and edge space foragers, such as the Leisler's bat and the common pipistrelle, when conspecific activity was low and playback buzz rate was high. In contrast to Daubenton's bats, Leisler's bats and common pipistrelles do not commonly focus on aquatic insects. Since low conspecific activity likely mirrors low prey availability, we assume that the (small) benefits of approaching these heterospecifics are only large enough to offset the costs of foraging on non‐preferred prey species in suboptimal open‐space habitats and of competing with potentially superior species, when the preferred prey is scarce but non‐preferred prey abundant. Similarly, open‐space foraging NEV bats approached sympatric *Myotis* species only when conspecific activity was very low and playback buzz rate very high. This result shows that NEV bats approach species of different feeding guilds only when very high buzz rates hint at large numbers of insects escaping the specialized foraging habitats of these *Myotis* species (water surface/clutter) into spheres where NEV bats can capture them.

High conspecific activity seems to pose high costs that negate all potential benefits of approaching conspecifics or heterospecifics for all species investigated. Even at high playback buzz rates, bat activity did not increase further if conspecific activity was already above a certain threshold. At very high conspecific activity, bats even inversed their responses and avoided the playback instead of approaching it in almost all cases. Our results thus suggest that activity reduction is a very wide‐spread response to high conspecific activity, performed by most insectivorous bats in response to various playback species even if very high buzz rates indicate large insect swarms. Most likely, high conspecific activity renders even dense prey clusters unprofitable due to pronounced intraspecific competition. Yet, increased competition for prey cannot be the only reason because bats also reduced activity when broadcasting calls from species of other foraging guilds that feed on different prey species than the eavesdropping bat. For instance, at high conspecific activity both trawling Daubenton's bats and aerial‐hawking common pipistrelles substantially reduced activity when we broadcast gleaning Natterer's bats. We assume that at high bat densities bats have to allocate considerable attention to collision avoidance and cannot focus on prey capture at the same time (Cvikel et al., 2015). Additionally, bats might suffer acoustic interference (“jamming”) when many individuals echolocate at close distance using similar frequencies (Gillam, Ulanovsky, & McCracken, 2007; but see Götze, Koblitz, Denzinger, & Schnitzler, 2016). At the same time, it is not a big loss to move from one foraging patch to another less busy one if prey is abundant there as well. Thus, any additional (simulated) bat can be too much to reside in the vicinity if conspecific activity is already high.

## 5 CONCLUSIONS

The community‐wide scale of our study enabled us to demonstrate that bats integrate social information about calling species, prey abundance and conspecific activity to fine‐tune their decision‐making. For the first time, we provide evidence that feeding buzz rates of some calling species profoundly affect the response of eavesdropping bats, and that these responses strongly depend on conspecific activity across all species investigated. These findings have major implications for the interpretation of previous studies and, especially, for our understanding of species interactions and coexistence. To date, studies investigating the eavesdropping behaviour of bats have not differentiated between different feeding buzz rates but commonly broadcast a single feeding buzz rate. Interpretations from these studies were then generalized, assuming that responses to the buzz rate presented would represent responses to any other buzz rate. Here, we show that such a generalization does not reflect the complexity of nature. Rather, bats made use of all relevant information they had access to when trading‐off costs and benefits of potential conspecific and heterospecific interactions to optimize their decisions. A comprehensive understanding of how bats incorporate social information into their decision‐making will help researchers explaining species distribution patterns and unravelling mechanisms of species coexistence (Gil et al., 2018). Furthermore, a detailed knowledge about the multifaceted intra‐ and interspecific interactions within bat communities is highly relevant for conservation measures and ecosystem management because eavesdropping on inadvertent social information can have demographic consequences and alter population as well as community dynamics (Gil et al., 2018). Particularly in human‐altered landscapes, eavesdropping can influence how populations respond to environmental change (Gil et al., 2018).

## AUTHORS’ CONTRIBUTIONS

H.R.G. conceived the idea and designed methodology together with D.L.; D.L. collected the data; D.L. and A.K.S. analysed the data; D.L. led the writing of the manuscript. All authors contributed critically to the drafts and gave final approval for publication.

## Data Availability

Data available from the Dryad Digital Repository: https://doi.org/10.5061/dryad.gp65g2t (Lewanzik, Sundaramurthy, & Goerlitz, 2019).
