# Supplementary material for: Insectivorous bats integrate social information about species identity, conspecific activity and prey abundance to estimate cost–benefit ratio of interactions
Source: J Anim Ecol. 2019 Apr 22;88(10):1462–73. doi: 10.1111/1365-2656.12989 (PMC6849779; doi:10.1111/1365-2656.12989)
Supplement: Supplementary file 1 [file JANE-88-1462-s001.pdf]

SUPPORTING INFORMATION

Insectivorous bats integrate social information about species identity, conspecific activity, and prey abundance to estimate cost-benefit ratio of interactions

Daniel Lewanzik[1], Arun K. Sundaramurthy[1,2], Holger R. Goerlitz[1]

[1]Acoustic and Functional Ecology, Max Planck Institute for Ornithology, Seewiesen
[2] Faculty of Biology, Ludwig-Maximilians-University, München

Authors for correspondence: hgoerlitz@orn.mpg.de & dlewanzik@orn.mpg.de

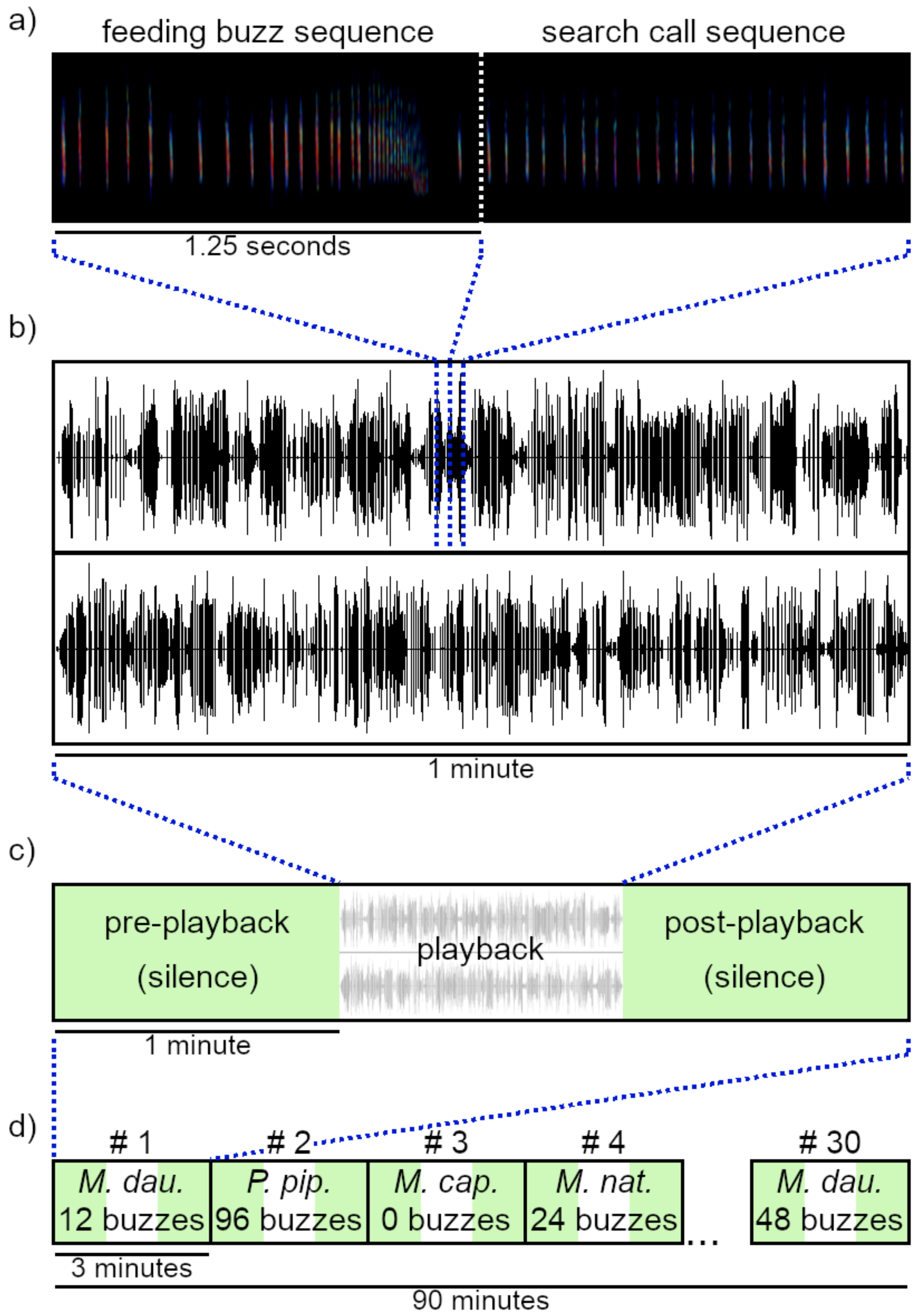

Fig. S1: Composition of playbacks. For each of six bat species (*Myotis capaccinii*, *M. daubentonii*, *M. nattereri*, *Nyctalus leisleri*, *Pipistrellus pipistrellus*, and *P. pygmaeus)* we combined 96 echolocation call sequences (each 1.25 s long; a) into 60 s long stereo playback files (b). Either 0, 12, 24, 48, or 96 of these short sequences contained a feeding buzz as shown in a); all other sequences contained only search calls. Buzz sequences were assigned pseudo-randomly to either of the two stereo channels, such that each channel contained n = buzz rate/2 buzz sequences embedded in consecutive search call sequences. As control we played an empty file of 1 min duration both before and after each 1-min playback file, such that in the end each playback block lasted 3 minutes and comprised three phases: pre-playback, playback, and post-playback, respectively (c). At each site we broadcast each playback species – buzz ratio combination only once in random order, resulting in 90 minutes (30 combinations x 3 min each) of playback (d).

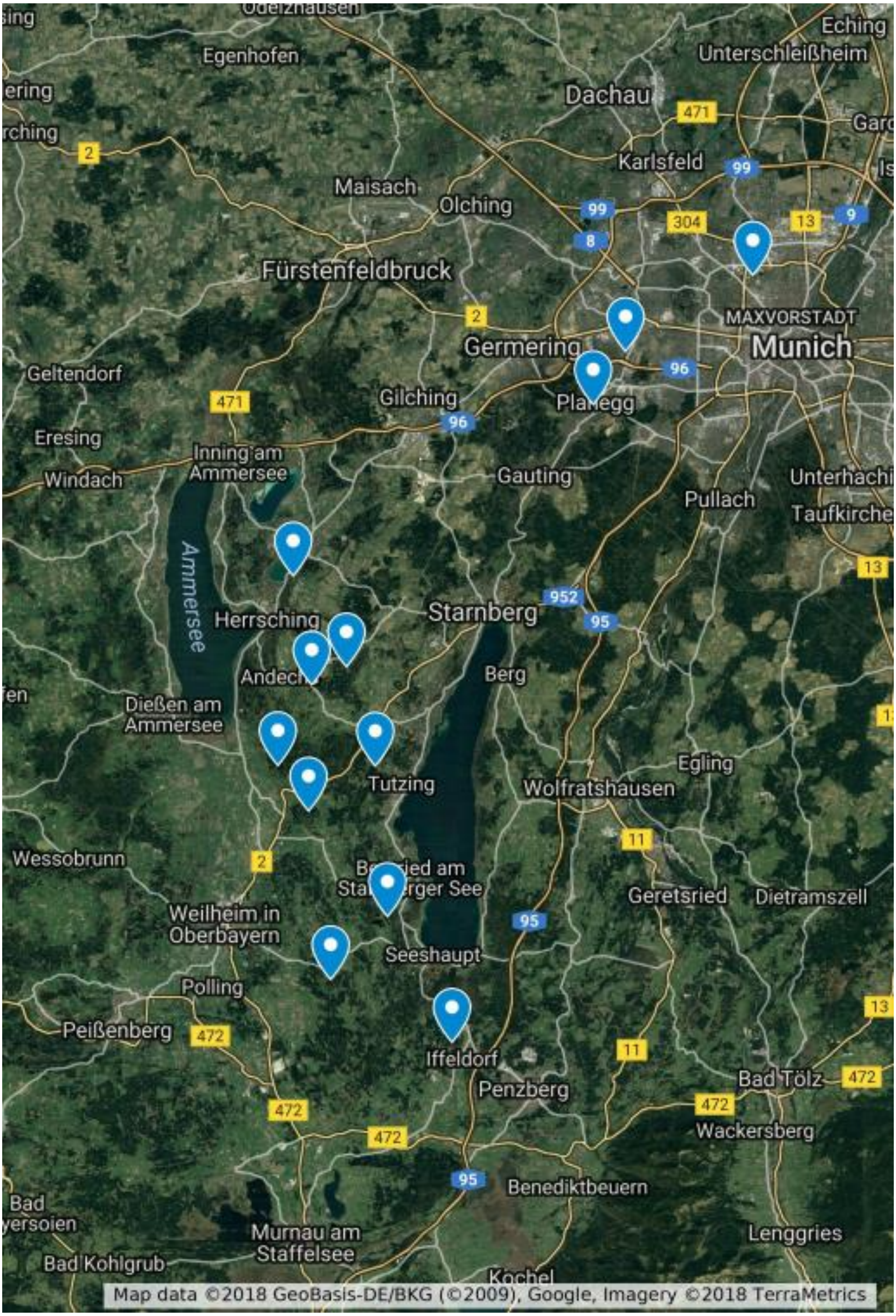

Fig. S2: Locations of 12 experimental sites in Southern Germany.

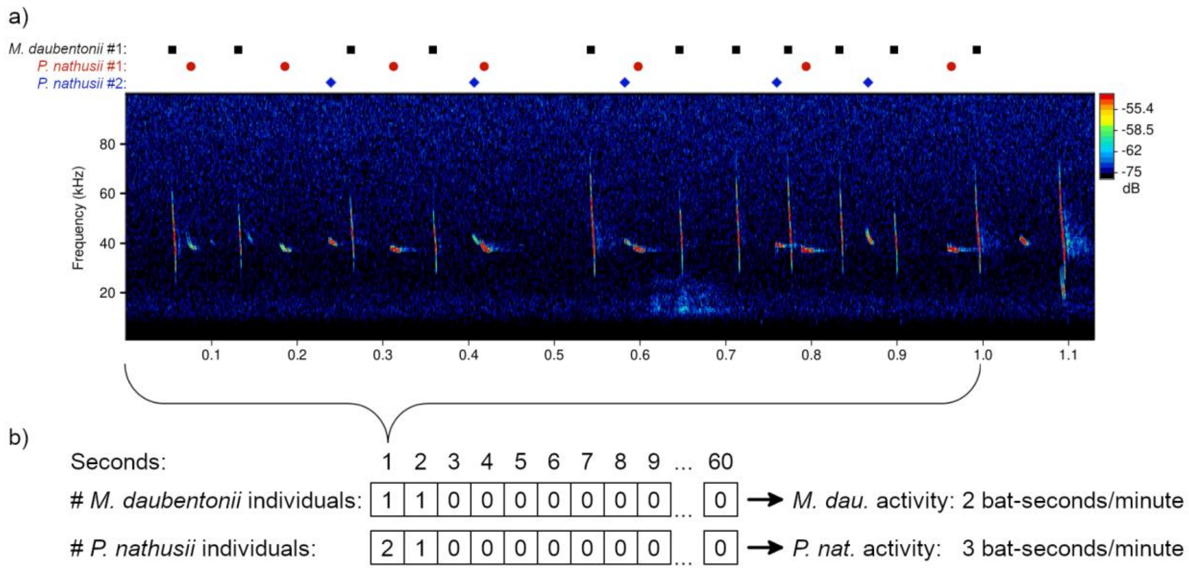

Fig. S3: Calculation of bat activity. For each recorded second we determined the number of individuals per species present based on frequency-time structure of calls and temporal patterns of call sequences. In the first second of this exemplary spectrogram (a) one *M. daubentonii* and two *P. nathusii* individuals were present (individual calls of each bat indicated by black squares, red dots, and blue diamonds, respectively). We summarised these counts in the boxes for the first second in c). By summing up these counts species-wise over all 60 seconds of each minute (assuming zero activity in seconds 3-60 in this example), we calculated species-specific activity as bat-seconds/minute (c).

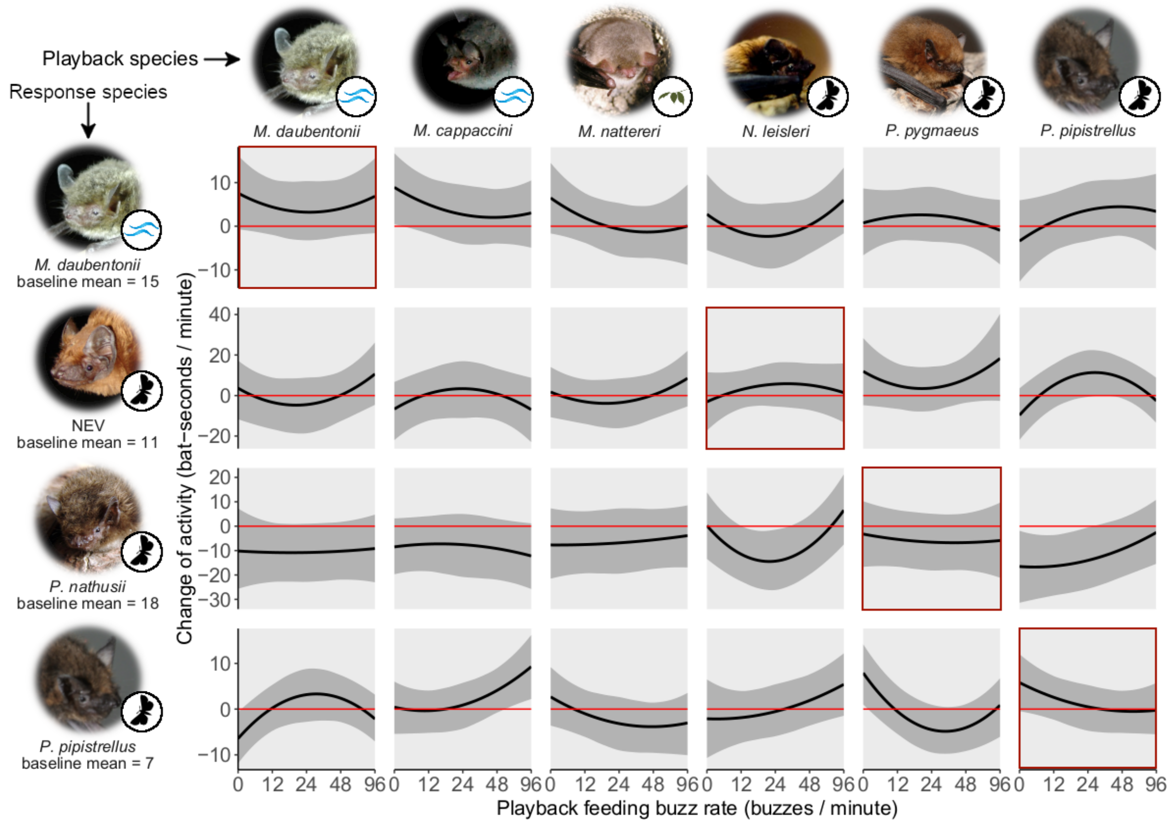

Fig. S4: Change of bat activity between control (silence) and treatment (search/buzz call playback) minutes for 24 combinations of playback and response species at species-specific mean conspecific activity (as indicated below pictures).

Conspecific activity is the actual bat activity in the control minute preceding a given treatment minute and was measured during data analysis. One-minute playbacks contained feeding buzzes embedded in search call sequences at rates of 0, 12, 24, 48, or 96 buzzes/min. Predictions and 95% CrIs (solid curved lines and shaded areas, respectively) are calculated for 1 hour after sunset, assuming no other species were present. Values above and below zero (horizontal red line) indicate activity increases and decreases, respectively. Red boxes highlight responses towards reference species. Symbols at pictures indicate main foraging modes: waves = trawling, leaves = gleaning, moth = aerial-hawking. Please note logarithmic x-scale and species-specific y-scales.

Photos: *M. nattereri* adapted from "FranseImWinterquartier" (© Guido Gerding, CC_BY-SA_3.0), *N. leisleri* adapted from "Nyctalus leisleri" (© Manuel Werner, CC_BY-SA_3.0), *P. nathusii* & NEV adapted from "Pipistrellus nathusii" & "Nyctalus noctula" (© Mnolf, CC_BY-SA_3.0), *P. pygmaeus* adapted from "Pipistrellus pygmaeus 01" (© Evgeniy Yakhontov, CC_BY-SA_3.0), *M. capaccinii* adapted from "Myotis capaccinii 0105b" (© Joxerra Aihartza, License Art Libre).

Table S1: Number of playback minutes analysed.

**response species**	**playback species**	**playback buzz rate (buzzes/min)**					
		0	12	24	48	96	sum
Myotis daubentonii	*M. daubentonii*	7	9	7	8	8	39
	M. capaccinii	8	8	7	10	9	42
	M. nattereri	8	9	10	10	6	43
	N. leisleri	7	5	8	10	9	39
	P. pipistrellus	7	10	10	6	7	40
	P. pyggmaeus	9	9	11	8	10	47
	sum	46	50	53	52	49	**250**
NEV group	*N. leisleri*	2	6	6	3	4	21
	M. capaccinii	3	3	3	2	2	13
	M. daubentonii	3	2	3	3	2	13
	M. nattereri	4	5	1	4	3	17
	P. pipistrellus	4	3	3	3	4	17
	P. pyggmaeus	2	3	4	3	1	13
	sum	18	22	20	18	16	**94**
Pipistrellus pipistrellus	*P. pipistrellus*	7	7	7	6	8	35
	M. capaccinii	7	6	8	7	5	33
	M. daubentonii	7	7	4	6	9	33
	M. nattereri	5	7	5	2	5	24
	N. leisleri	3	5	6	4	5	23
	P. pyggmaeus	6	5	7	7	7	32
	sum	35	37	37	32	39	**180**
P. nathusii	*P. pyggmaeus*	5	7	5	2	3	22
	M. capaccinii	5	2	4	3	5	19
	M. daubentonii	2	3	4	3	4	16
	M. nattereri	3	3	3	4	3	16
	N. leisleri	3	4	6	4	3	20
	P. pipistrellus	3	3	3	4	4	17
	sum	21	22	25	20	22	**110**
